# Review of 21 cases of mycetoma from 1991 to 2014 in Rio de Janeiro, Brazil

**DOI:** 10.1371/journal.pntd.0005301

**Published:** 2017-02-13

**Authors:** Felipe Maurício Soeiro Sampaio, Bodo Wanke, Dayvison Francis Saraiva Freitas, Janice Mery Chicarino de Oliveira Coelho, Maria Clara Gutierrez Galhardo, Marcelo Rosandiski Lyra, Maria Cristina da Silva Lourenço, Rodrigo de Almeida Paes, Antonio Carlos Francesconi do Valle

**Affiliations:** National Institute of Infectious Diseases, Oswaldo Cruz Foundation - Rio de Janeiro - Brazil; University of California San Diego School of Medicine, UNITED STATES

## Abstract

Mycetoma is caused by the subcutaneous inoculation of filamentous fungi or aerobic filamentous bacteria that form grains in the tissue. The purpose of this study is to describe the epidemiologic, clinic, laboratory, and therapeutic characteristics of patients with mycetoma at the Oswaldo Cruz Foundation in Rio de Janeiro, Brazil, between 1991 and 2014. Twenty-one cases of mycetoma were included in the study. There was a predominance of male patients (1.3:1) and the average patient age was 46 years. The majority of the cases were from the Southeast region of Brazil and the feet were the most affected anatomical region (80.95%). Eumycetoma prevailed over actinomycetoma (61.9% and 38.1% respectively). Eumycetoma patients had positive cultures in 8 of 13 cases, with isolation of *Scedosporium apiospermum* species complex (n = 3), *Madurella mycetomatis* (n = 2) and *Acremonium* spp. (n = 1). Two cases presented sterile mycelium and five were negative. Six of 8 actinomycetoma cases had cultures that were identified as *Nocardia* spp. (n = 3), *Nocardia brasiliensis* (n = 2), and *Nocardia asteroides* (n = 1). Imaging tests were performed on all but one patients, and bone destruction was identified in 9 cases (42.68%). All eumycetoma cases were treated with itraconazole monotherapy or combined with fluconazole, terbinafine, or amphotericin B. Actinomycetoma cases were treated with sulfamethoxazole plus trimethoprim or combined with cycles of amikacin sulphate. Surgical procedures were performed in 9 (69.2%) eumycetoma and in 3 (37.5%) actinomycetoma cases, with one amputation case in each group. Clinical cure occurred in 11 cases (7 for eumycetoma and 4 for actinomycetoma), and recurrence was documented in 4 of 21 cases. No deaths were recorded during the study. Despite of the scarcity of mycetoma in our institution the cases presented reflect the wide clinical spectrum and difficulties to take care of this neglected disease.

## Author summary

Mycetoma is a major health problem in tropical areas and is prevalent among people of low socio-economic status. As in many other regions of the world, the incidence and prevalence of mycetoma in Brazil is unknown. This study describes some aspects of mycetoma patients in 24 years of experience at the National Institute of Infectious Diseases at the Oswaldo Cruz Foundation, Rio de Janeiro, Brazil and contribute to the knowledge on mycetoma epidemiology globally.

## Introduction

Mycetoma is a chronic subcutaneous infections caused by the inoculation of filamentous fungi (eumycetoma) or aerobic filamentous bacteria (actinomycetoma) that form grains in the affected tissues [1]. It´s considered a neglected disease by the World Health Organization (WHO) since 2016 and remains without any control program for prevention or surveillance [1, 2].

Mycetoma occurs worldwide and prevails in tropical and subtropical regions, especially in sub-Saharan areas of Africa, India, and Mexico [3,4]. In South America, cases have been reported in Venezuela, Colombia, Brazil, and Argentina [1,3,5]. The incidence and prevalence of mycetoma in Brazil are unknown, since it is not considered a public health problem, as its frequency is smaller than other diseases such as sporotrichosis, tuberculosis, leprosy, and dengue (the latter two are classified as neglected diseases by the WHO) [6]. Mycetoma evolves slowly in its clinical manifestation. Laboratory diagnosis and treatment are difficult, presenting significant medical, occupational and socioeconomic impacts [2,7].

In this study, we describe the epidemiological, clinical, laboratory, and therapeutic aspects of patients treated at a reference hospital in Rio de Janeiro, Brazil, between 1991 and 2014.

## Methods

### Ethics

The study was approved by the Research Ethics Committee of the INI / Fiocruz, on November 25, 2013 under the number 477.037. All participants gave their written consent, with the exception of those who died before the study. In all cases the identity and information of each patient were preserved.

### Laboratorial diagnosis

Histological examination was performed using haematoxylin-eosin, Grocott’s methenamine silver, Periodic acid–Schiff, and Gram-Brown-Brenn stains. Biopsy specimens were submitted for direct microscopic examination with 10% potassium hydroxide where grains were classified according to their size, shape, colour, consistency and presence of hyphae or filamentous bacteria. Culture on Sabouraud's Dextrose Agar 2% and Mycobiotic Agar was performed for eumycotic grains and in/on Lowenstein-Jensen medium, defibrinated sheep blood agar chocolate agar and thioglycolate medium with resazurina for actinomycotic grains.

Bacterial and fungal etiologic agents were identified by examination of the colonies in culture.

### Imaging tests

Ultrasound, radiography, computerized tomography (CT), and magnetic resonance imaging (MRI) were performed to determine deep tissue and bone involvement and presence of grains.

### Treatment

Actinomycetoma patients were treated with oral sulfamethoxazole-trimethoprim (SMX-TMP) 800/160 mg BD, alone or in combination with alternate cycles of 15 mg/kg/day intravenous amikacin for three weeks in cases with bone destruction. Other antimicrobials were given in case of secondary infections. Eumycetoma patients were treated with itraconazole (200 mg, BD) alone or, in cases without consistent clinical response after six months, in combination with fluconazole 200 mg/day, terbinafine 250 mg/day, or amphotericin B 1mg/kg. Surgical treatment was indicated for small and delimited lesions and in cases of bone destruction. Amputation was indicated in cases lacking a satisfactory antimicrobial response associated to severe bone destruction of the affected segment.

### Follow-up

The patients were followed-up bimonthly at the outpatient clinic to assess clinical responses to treatment and drug side effects. A complete cure was defined with the healing of lesions, bone remodelling, and absence of grains upon imaging examination. After the determination of the clinical cure, outpatient follow-up turned annual, to assess the possibility of recurrence.

### Statistics

Data retrieved from patients records were analysed using descriptive statistics with the Statistical Package for the Social Sciences, version 20.0. Data were summarized as percentages for categorical variables and mean, median, and range for continuous variables.

## Results

A total of 21 mycetoma cases were included in the present study: 13 eumycetoma and 8 actinomycetoma patients.

The main sociodemographic aspects of the mycetoma patients are summarised in Table 1. In brief, the male to female ratio was 1.3:1, and the mean age was 46 years old (range 28–93 years). However, the mean age for eumycetoma was 51.3 years old and 38.6 years old for actinomycetoma. The non-white ethnicity/race predominated with 66,66%. Most patients (71.43%) came from the southeast region of Brazil, and 28,57% came from the northeast region. These regions correspond to the possible original infection sites.

**Table 1:** Demographic, clinical and laboratorial characteristics of 21 patients diagnosed with mycetoma at the Evandro Chagas National Institute of Infectious Diseases, Oswaldo Cruz Foundation, Rio de Janeiro—Brazil, from 1991 to 2014.

Demographic characteristics	Number	Percentage
**Gender**		
** Male**	12	57.14
** Female**	9	42.85
**Ethnicity/color**		
** White**	7	33.33
** Non-white**	14	66.66
**Geographic Origin**		
** Southeast Brazil**	15	71.42
** Northeast Brazil**	6	28.57
**Age (years)**		
** < 30**	3	14.28
** 30–50**	9	42.85
** > 50**	9	42.85
		
**Comorbidity**		
		
**Systemic hypertension**	7	33.33
**Diabetes Mellitus**	3	14.28
**HIV**	1	4.76
**Asthma**	1	4.76
**None**	11	52.38
		
**Anatomical regions**		
** Foot**	17	80.95
** Ankle**	1	4.76
** Hand**	1	4.76
** Thighs and buttocks**	2	9.52
**Bone involvement**		
** Yes**	9	42.85
** No**	12	57.14

HIV: human immunodeficiency virus.

* Some patients have more than one comorbidity.

**Fig 1:**
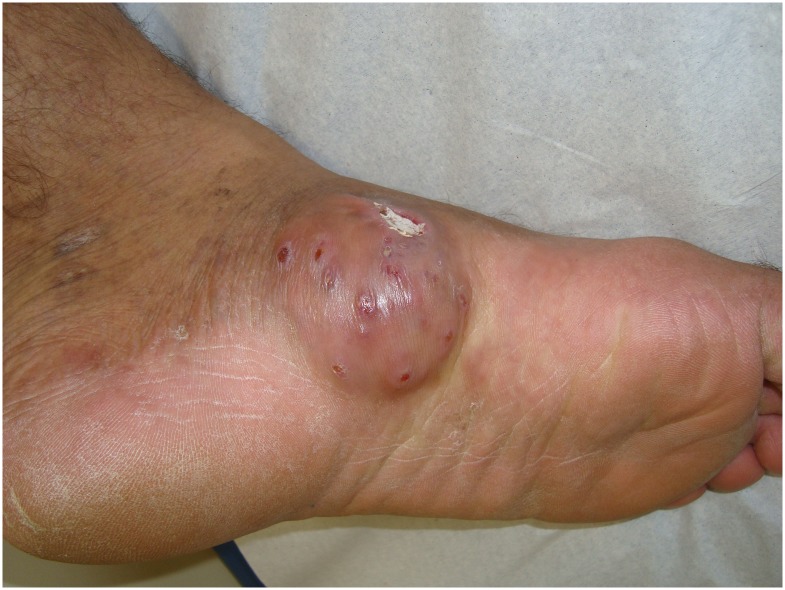
HIV patient with actinomycetoma before treatment.

**Fig 2:**
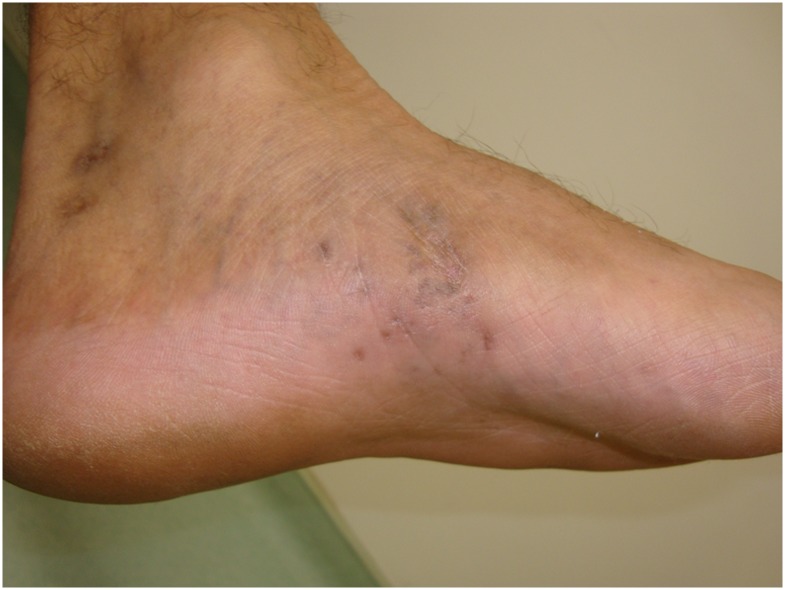
HIV patient with actinomycetoma after treatment.

Comorbidities occurred in 10 patients. Eight of them presented a single comorbidity and the others had two comorbidities. In general, high blood pressure, diabetes mellitus, HIV positive (Figs 1 and 2), and asthma were found.

The time from onset of signs and symptoms to medical care ranged from 2 to 420 months (mean = 77.68 months). The average time was higher for eumycetoma (mean = 105.76 months) than actinomycetoma (mean = 36.75 months).

**Fig 3:**
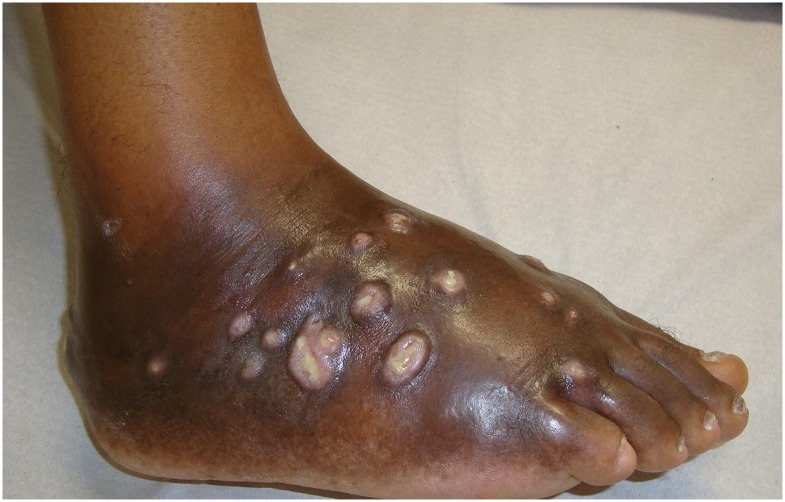
Foot affected with actinomycetoma.

**Fig 4:**
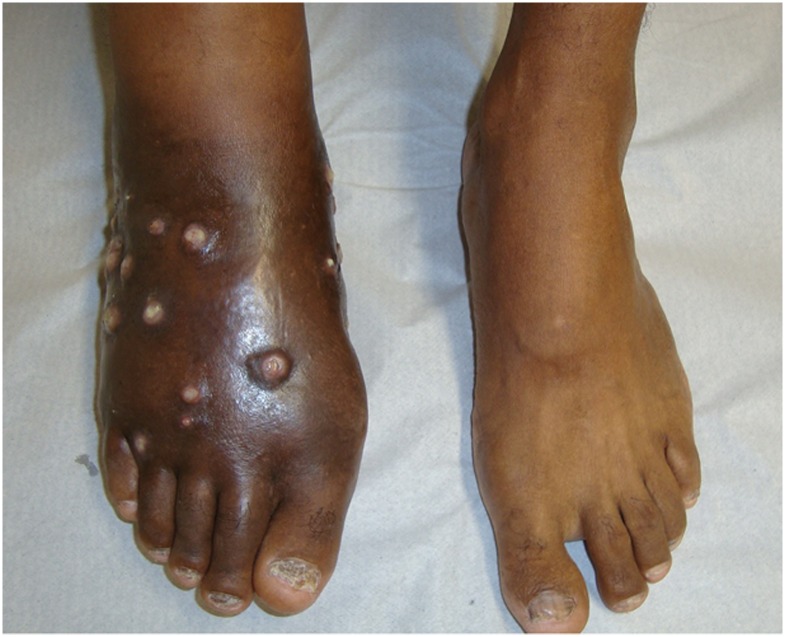
Right foot affected with actinomycetoma and the left foot without disease.

**Fig 5:**
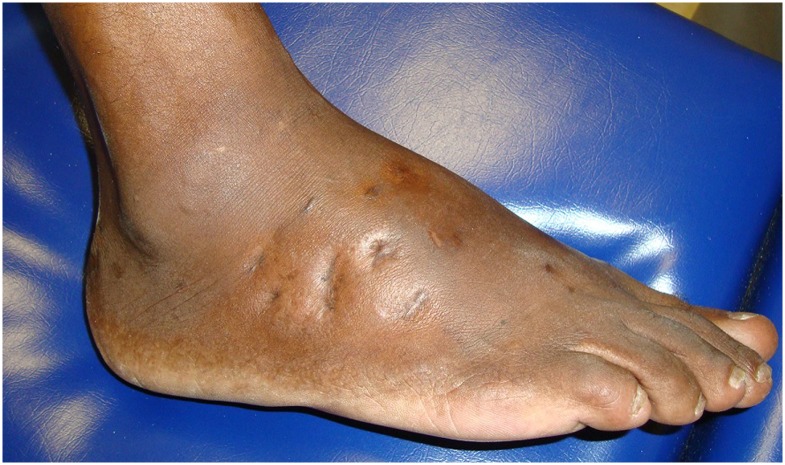
Right foot affected with actinomycetoma after treatment.

The foot (Figs 3, 4 and 5) was affected in 17 cases (80.9%), the thigh was affected in two cases, and the hand and ankle were affected in one case each. A history of trauma was reported in 17 (80.9%) cases.

The grains were mainly identified through histopathological examination with 90.4% positivity in these methods and 9.6% through direct microscopy. We retrieved the etiological agents in 61.5% eumycetoma cases (Table 2) and in 75% of actinomycetoma cases (Table 3).

**Table 2:** Main characteristics of 13 patients with Eumycetoma.

Patient	Sex	Etiologic agent	Grains	Treatment	Time for treatment (months)	Surgery	Bone involvement	Outcome
1	F	*Madurella mycetomatis*	H	ITZ / AMB	24	Yes*	Yes	Cure
2	M	*Madurella mycetomatis*	H/DM	ITZ + FLZ	60	Yes	No	Cure
3	M	*Acremonium* sp.	H	ITZ + FLZ	144	Yes	Yes	No Cure
4	M	*Scedosporium apiospermum*	H/DM	ITZ	46	Yes	No	Cure
5	M	*Scedosporium apiospermum*	H/DM	ITZ + TBF	**	No	No	**
6	M	*Scedosporium apiospermum*	H	ITZ + TBF / ITZ + FLZ	74	No	Yes	***
7	M	Filamentous fungi	H	ITZ	123	Yes	No	Cure
8	F	Filamentous fungi	H/DM	ITZ	**	No	Yes	**
9	F	Negative culture	H/DM	ITZ	24	Yes	No	Cure
10	F	Negative culture	H	ITZ	36	Yes	Yes	No cure
11	M	Negative culture	H	ITZ	7	Yes	No	Cure
12	F	Negative culture	H	ITZ	9	Yes	No	Cure
13	M	Negative culture	H	ITZ	6	No	No	***

F: female; M: male

H: histopathology; DM: direct microscopy; ITZ: itraconazole; FLZ: fluconazole; TBF: terbinafine; AMB: amphotericin B.

*Amputation;

** Abandon of follow up;

*** Still in treatment.

**Table 3:** Main characteristics of 8 patients with Actinomycetoma.

Patient	Sex	Etiologic agente	Grains	Treatment	Time for treatment (months)	Surgery	Bone involvement	Outcome
1	M	*Nocardia brasiliensis*	H	TMP + SMX	48	Yes	Yes	***
2	M	*Nocardia brasiliensis*	H/ DM	TMP + SMX	38	No	Yes	**
3	F	*Nocardia* sp.	DM	TMP + SMX	36	No	No	Cure
4	M	*Nocardia* sp.	DM	TMP + SMX	10	No	No	Cure
5	M	*Nocardia* sp.	H	TMP + SMX	8	No	No	***
6	F	*Nocardia asteroides*	H	TMP + SMX / cefalexin	19	Yes *	Yes	Cure
7	F	Negative culture	H	—	0	Yes	No	Cure
8	F	Negative culture	H	TMP + SMX/ 5 cycles of amikacine	24	No	Yes	***

F: female; M: male

H: histopathology; DM: direct microscopy; TMP + SMX: Trimethoprim/sulfamethoxazole.

* Amputation;

** Abandon of follow up;

*** Still in treatment.

In the eumycetoma group, the *Scedosporium apiospermum* species complex was identified in three cases, *Madurella mycetomatis* was isolated from two cases and *Acremonium* sp. was isolated from one case. From the remaining two patients, the isolated filamentous fungi could not be identified, as they only produced hyphae without any conidia or spores, and therefore they were named Mycelia sterilia (cases 7 and 8, Table 2). It is important to note that these two organisms were consistently isolated as pure cultures in at least three consecutive mycological examinations.

In the actinomycetoma group we isolated *Nocardia* spp. from three cases, *Nocardia brasiliensis* from two cases and *Nocardia asteroides* from one case (Table 3). In two cases the culture were negative.

**Fig 6:**
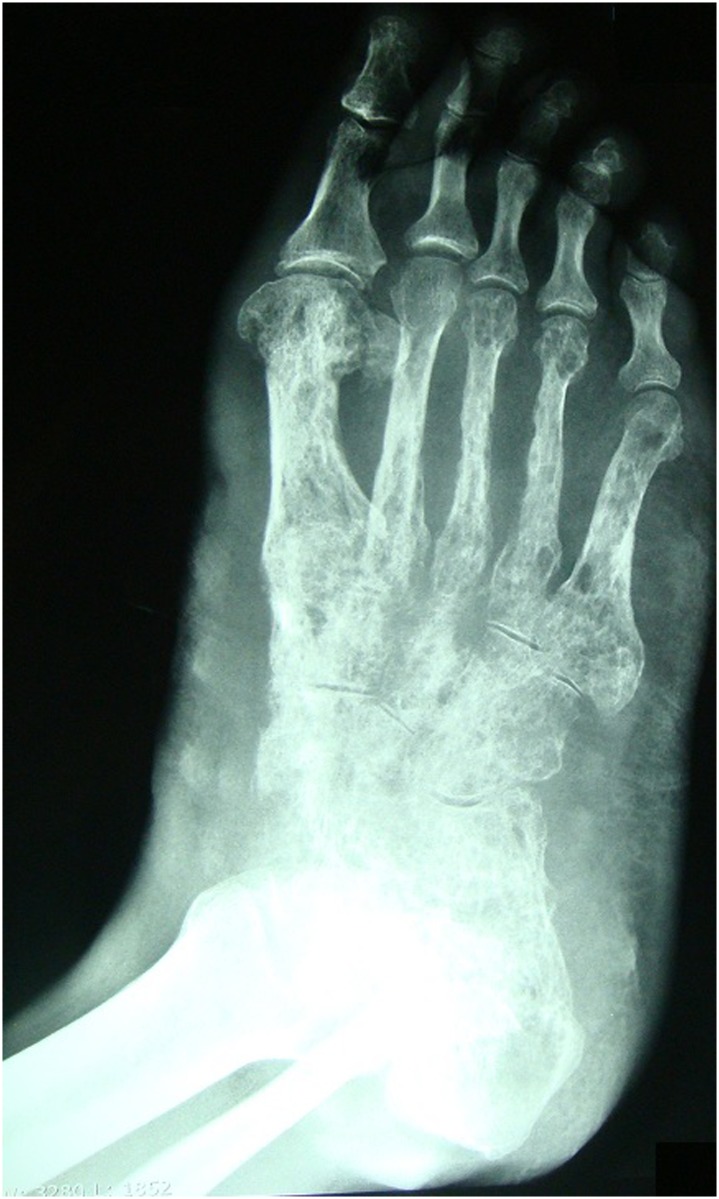
Radiography showing bone destruction.

All patients underwent radiography of the affected site with exception of patient 7 of Table 3, who underwent complete excision with security margin of the lesion during the diagnostic procedure (Fig 6).

Ultrasonography was performed in 18 cases, with the observation of subcutaneous nodules in all of them. Ten patients underwent CT scans and seven patients underwent MRI. Bone involvement was present in 9 cases, five from eumycetoma and four from actinomycetoma.

Secondary bacterial infection was diagnosed in four cases, two of them had *Staphylococcus aureus* associated infection treated with systemic antibiotics guided by susceptibility tests. The other two cases were treated empirically.

Patients with eumycetoma received 200 mg BD itraconazole alone (8/13) or in combination with 200 mg/day fluconazole (3/13), or 250 mg/day terbinafine (2/13). In case 1 (Table 2), when the patient became pregnant during itraconazole treatment, this drug was suspended and we tried to use liposomal amphotericin B due to clinical worsening, without success.

Actinomycetoma patients received 800/160 mg sulfamethoxazole-trimethoprim BD in most of cases (75%). As monotherapy in five cases, one case with cycles of 15 mg/kg/day amikacin sulphate and another case received 500 mg cephalexin four times a day. The used of cephalexin occurred because of the repeated secondary bacterial infection. The case 7 (Table 3) with a small and well delimited lesion in lower limb underwent complete excision with security margin and therefore was not treated with antimicrobials. The case 8 (Table 3), who presented with multiple foci of bone destruction, was submitted to amikacin cycles, which had to be stopped after the fifth cycle due to changes in audiometry and increased creatinine levels, without lifelong clinical consequences. Itraconazole was used in all cases and combined with another antifungal agents (38%) in refractory cases.

The average treatment time was 35.04 months (range 6–144 months). The mean treatment time was 42.53 months for eumycetoma and 22.87 months for actinomycetoma. The average treatment time for patients with bone destruction was 70 months (median 55 months) for eumycetoma cases and 33 months (median 36 months) for actinomycetoma cases.

Amputation was recommended for three patients with eumycetoma, one of them accepted the procedure and the other two remain receiving drug treatment until now. In the actinomycetoma group, one patient accepted amputation. Surgical excision of small lesions were performed in nine eumycetoma patients and three actinomycetoma patients.

Clinical cure occurred in 11 (52.38%) of all cases. Of the 13 eumycetoma patients, seven were cured, two abandoned follow up and another two patients are still under antifungal treatment. Of the five eumycetoma patients with bone involvement, one underwent amputation, two remained in treatment, one remain under observation and one abandoned treatment.

Of the eight actinomycetoma patients, four were cured, one abandoned the treatment and three are under treatment. Of the four actinomycetoma cases with bone involvement, one patient was underwent amputation, two remained in treatment and one abandoned treatment. If we consider the cure without sequelae (amputation), the rate falls to 42.8%.

Recurrence of infection was observed in four patients: one with actinomycetoma and three with eumycetoma. The time to recurrence was 24 months for the actinomycetoma case and ranged from 8 to 96 months (mean = 36.6 months) for eumycetoma cases.

Treatment dropouts was high (23%) and recurrence was also frequent (19%) and prevailed in patients that had undergone surgery, especially in the eumycetoma group.

The broad range of treatment duration until clinical cure (6–114 months) was a striking observation of this study.

## Discussion and review of the literature

The 21 mycetoma cases diagnosed in the 24-year period of this study demonstrate the low frequency of mycetoma in our institution at Rio de Janeiro, Brazil. Most reports of mycetoma in Brazil describe one or a few cases, reinforcing the scarcity of the disease in this country. To achieve a better comprehension on this subject we performed a search of articles on PubMed (from 1980 to 2014) using the MESHterms “Mycetoma”, “Actinomycetoma”, and “Eumycetoma” alone or in combination with “Brazil”. During this period, 272 mycetoma cases were reported (Table 4). This number is smaller than that observed in Sudan and Mexico [8, 9,10]. For instance, in Mexico, where 483 mycetoma cases were diagnosed at a single hospital during the same period [11]. In 2013, van de Sande et al. [1] estimated the prevalence of mycetoma cases in Mexico and the Sudan as 0.15 and 1.81 cases per 100,000 inhabitants, respectively, compared to the prevalence of less than 0.001 per 100,000 inhabitants in Brazil.

**Table 4:** Mycetoma cases in Brazil published during 1980–2014.

Reference	Year	Study	Classification	Number of cases	Etiologic agent	Region
[73]	1980	Clinical report + review	unknown	4	Unknow	Amazonas (North)
[49]*	1980	Clinical report	Eumycetoma	1	*Petriellidium boydii (Scedosporium apiospermum)*	-
[50]	1981	Clinical report	Eumycetoma	1	*Petriellidium boydii (Scedosporium apiospermum)*	Minas Gerais (Southeast)
[12]	1981	Retrospective (1944–1978)	▪Eumycetoma ▪Actinomycetoma	41 113	▪Unknown (Histopathological aspects) ▪**** Review (1916–1980): 26 cases	Unknow
[27]	1982	Clinical report	Actinomycetoma	6	*Nocardia* sp.	São Paulo (Southeast)
[39]*	1982	Clinical report	Eumycetoma	1	*Madurella grisea*	-
[35]	1984	Clinical report + review	Actinomycetoma	1	▪*Actinomadura madurae* ▪**** Review 10 cases	São Paulo (Southeast)
[74]	1986	Case series	Actinomycetoma	4	*Nocardia brasiliensis*	Rio Grande do Sul (South)
[75]	1988	Retrospective	Unknown	2	Unknow	Amazonas (North)
[46]	1988	Clinical report + review	Eumycetoma	1	▪*Acremonium falciforme* ▪**** Review 6 cases	Bahia (Northeast)
[52]*	1988	Clinical report	Eumycetoma	1	*Exophiala jeanselmei*	-
[40]*	1989	Clinical report	Eumycetoma	1	*Madurella grisea*	-
[41]	1989	Clinical report	Eumycetoma	1	*Madurella grisea*	-
[76]	1990	Comunication	Actinomycetoma	1	Unknow	Ceará (Northeast)
[42]	1991	Clinical report	Eumycetoma	1	*Madurella grisea*	Goiás (Midwest)
[77]*	1991	-	-	-	-	Goiânia-Goiás (Midwest)
[13]	1992	Clinical report + review	Actinomycetoma	2	▪*Actinomadura madurae* ▪**** Review: Actinomicetoma 61 cases Eumicetoma 33 cases	Rio de Janeiro (Southeast) Pernambuco (Northeast)
[43]	1992	Clinical report	Eumycetoma	2	▪*Madurella grisea* ▪**** Review 6 cases	Bahia (Northeast)
[33]	1993	Clinical report	Actinomycetoma	1	*Nocardia asteroides*	Rio de Janeiro (Southeast)
[14]	1993	Retrospective 1978–1989	▪Eumycetoma ▪Actinomycetoma	13 28	▪*Madurella grisea—*3 ▪*Scedosporium apiospermum—*2 ▪*Madurella mycetomatis—*1 ▪Culture negative—7 ▪*Nocardia brasiliensis—*13 ▪*Actinomadura madurae—*1 ▪*Actinomadura pelletieri—*1 ▪*Nocardia asteroides—*1 ▪Culture negative—12	Northeast Southeast South
[37]	1993	Clinical report	Eumycetoma	2	*Madurella mycetomatis*	Bahia and Piauí(Northeast)
[26]	1994	Clinical report + review	Actinomycetoma	1	▪*Nocardia brasiliensis* ▪**** Review: 1954–1990: 26 cases	Pará (North)
[28]	1995	Clinical report	Actinomycetoma	1	*Nocardia brasiliensis*	Minas Gerais (Southeast)
[18]	1999	Clinical report	Actinomycetoma	1	Unknow	São Paulo (Southeast)
[44]	1999	Clinical report	Eumycetoma	1	*Exophiala jeanselmei*	Rio Grande do Sul (South)
[47]	1999	Clinical report	Eumycetoma	1	*Acremonium kiliense*	Bahia (Northeast)
[78]	1999	Clinical report	Eumycetoma	1	▪*Madurella grisea* ▪**** Review 8 cases	Rio Grande do Sul (South)
[51]	2002	Clinical report	Eumycetoma	1	*Fusarium solani*	São Paulo (Southeast)
[29]	2004	Clinical report	Actinomycetoma	1	*Nocardia brasiliensis*	Minas Gerais (Southeast)
[45]	2004	Clinical report	Eumycetoma	1	▪*Madurella grisea* ▪**** Review 11 cases	Rondônia (North)
[30]	2007	Case series	Actinomycetoma	1	*Nocardia brasiliensis*	Rio Grande do Sul (South)
[15]	2008	Retrospective	▪Eumycetoma ▪Actinomycetoma	13 14	▪*Madurella mycetomatis—*3 ▪*Madurella grisea—*1 ▪*Acremonium kiliense—*1 ▪*Acremonium* sp.—1 ▪Culture negative—7 ▪*Nocardia brasiliensis—*3 ▪*Nocardia asteroides—*1 ▪*Streptomyces somaliensis—*1 ▪Culture negative—9	Southeast Northeast Southeast Northeast
[36]	2010	Clinical report	Actinomycetoma	1	*Actinomadura madurae*	Paraíba (Northeast)
[34]**	2010	Clinical report	Actinomycetoma	1	*Nocardia caviae (Nocardia ottitidiscaviarum)*	Minas Gerais (Southeast)
[31]	2011	Clinical report	Actinomycetoma	1	*Nocardia brasiliensis*	Northeast
[79]	2011	Clinical report	Eumycetoma	1	-	São Paulo (Southeast)
[53]	2011	Clinical report	Eumycetoma	1	*Exophiala jeanselmei*	Paraná (South)
[38]***	2013	Clinical report	Eumycetoma	1	*Madurella mycetomatis*	Ceará (Northeast)
[48]	2013	Clinical report + review	Eumycetoma	1	*Scedosporium apiospermum*	Rio Grande do Sul (Southeast)
[32]	2014	Clinical report	Actinomycetoma	1	*Nocardia brasiliensis*	São Paulo (Southeast)
[25]***	2014	Clinical report	Eumycetoma	1	*Madurella mycetomatis*	Rio de Janeiro (Southeast)

* Article with reference but not found.

** Article with doubt about the diagnostic proposed by the author because he has not reported the existence of grain.

*** Case report inside the study.

**** Review of the author.

The predominance of eumycetoma in our study might not represent the real scenery of mycetoma in Brazil, as the Brazilian literature reveals a higher frequency of actinomycetoma (Table 4) [12,13,14,15]. The involvement of male individuals above 30 years old with an acral location likely due to increased risk exposure during labour activity without safety equipment is in accordance with mycetoma characteristics [5,16,17].

Although eumycetoma and actinomycetoma share similar clinical aspects, we noted that eumycetoma cases usually tend to be more silent and chronic, while actinomycetoma cases were more inflammatory and painful. This fact may explain why patients with eumycetoma take longer to seek medical care.

We noted that six of our patients moved from the Northeast region of Brazil to the Rio de Janeiro state, in the Southeast region, probably attracted for job possibilities in a state with higher socio-economic index, higher urbanization of population and better health infrastructure. For this reason, we assume that, for these patients, the place where infection occurred was not in Rio de Janeiro.

Comorbidities are not associated to more severe or atypical forms of mycetoma and there are no changes in the course of mycetoma in the HIV infected patient [18,19,20]. Although it requires further investigation, pregnancy may be linked to more severe clinical course of mycetoma [21,22,23,24] as in case 1 (Table 2) that developed severe bone destruction during pregnancy, resulting in amputation of the affected limb [25].

The mycetoma agents identified in our study are consistent with previous reports. In the actinomycetoma group, *Nocardia* spp., particularly *N*. *brasiliensis*, predominated and in the eumycetoma group, *Scedosporium apiospermum*. From 1980 to 2014, the main bacterial agents identified in Brazil were *Nocardia brasiliensis* [15,26,27–32], *Nocardia asteroides* [15,33], *Nocardia caviae* [34], *Actinomadura madurae* [13,35,36], *Actinomadura pelletieri* [14], and *Streptomyces somaliensis* [15]. For eumycetoma were *Madurella mycetomatis* [15,25,37,38], *Madurella grisea* [39–45], *Acremonium falciforme* [46], *Acremonium kiliense* [47], *Scedosporium apiospermum* [12,18,48,49,50], *Fusarium solani* [51], *Exophiala jeanselmei* [44,52,53] and *Aspergillus* sp. [12].

In our series of cases the diagnosis of mycetoma was made mainly by histopathological examination of affected tissues with visualization of the grains (approximately 91% of cases), while the isolation of the etiologic agent by culture was obtained in 66.6% of cases [15]. Implementation of molecular tools have recently demonstrated an improvement in the sensitivity and specificity in diagnosing mycetoma [16].

Radiography and ultrasonography were the most often used imaging because of their low cost and accessibility. Ultrasonography was crucial in identifying the presence of grains before diagnosis, during and after the therapeutic follow-up. Magnetic resonance imaging is the gold standard imaging method for mycetoma diagnosis and was important to delineate the involvement of internal structures and surgical planning [54]. CT scan was used if no bone involvement was detected by radiography.

Mycetoma treatment is challenging and usually requires long periods of drug therapy with or without surgical procedures (complete excision of the lesion, bone curettage, amputation) [1,5,8,10]. Itraconazole is the most common antifungal agent used for eumycetoma treatment [2]. Voriconazole and posaconazole have been indicated for refractory cases of mycetoma [58] primarily caused by *S*. *apiospermum* and *Acremonium* sp. [48,59–62]. They are expensive in underdeveloped countries and are not available in our institution. Isavuconazole and ravuconazole seem to be satisfactory against *M*. *mycetomatis* [63,64] but their effectiveness against other eumycetoma agents need to be investigated.

The first patient in this series of cases was evaluated in 1991, and because of this, the combination of drugs used was based on the available drugs at that time in our institution. The combined itraconazole/fluconazole, and itraconazole/terbinafina treatment in this study was chosen because of our good experience in treating extensive cutaneous lesions of chromoblastomycosis caused by *Fonsecaea pedrosoi* [55]. However, currently the itraconazole/fluconazole combination for mycetoma is not effective. Although liposomal amphotericin B are no longer recommended for first-line eumycetoma treatment, due to the high minimum inhibitory concentrations required for most eumycetoma agents [16,17,21,56,57], we tried to use only in one case due to clinical worsening during pregnancy, without success [25].

The recommended treatment for actinomycetoma is SMX/TMP as monotherapy or in combination with amikacin sulphate [10]. The association usually gives a cure rate above 90% [2, 65, 66]. Laboratory tests are required to assess possible adverse effects, as ototoxicity (cochlear lesions) and nephrotoxicity, which are permanent injuries, but are not progressive when treatment is suspended. In case 8 of Table 3 a combination with amikacin sulphate was used due to bone destruction. Amoxicillin and clavulanate are alternative drugs during pregnancy, for resistant cases or for patients with adverse effects from aminoglycoside [3]. Rifampicin can be used, but in Brazil it is reserved for tuberculosis and leprosy treatment, diseases with a high burden in our country. Minocycline and moxifloxacin are also treatment options for actinomycetoma [2, 67].

Surgery is indicated for small well localised lesions or in patients who are not responding to medical therapy or to reduce disease burden in massive lesions to allow a better response to medical therapy. [68]. Usually, actinomycetoma require less surgery management then eumycetoma [10]. Amputation are indicated for those patients with massive disease with no response to medical treatment or with massive bone destruction or in case with severe secondary bacterial infection not responding to medical treatment or with severe drug side-effects. [3]

Although our institution has provided all antimicrobials necessary for the treatment free of cost to all patients, the cure rate in this study was low, which reflects the difficulties in treating this disease. Besides the inconvenience to take pills every day for a long period, the total cost of mycetoma treatment is unaffordable for people living in poor regions where the disease commonly occurs. We suggest that the low rate of cure in our study is multifactorial, including the delay to obtain a correct diagnosis, and the scarcity of specialized surgical services with knowledge about this disease that allow the management of the most advanced cases. The postponement of diagnosis favours the occurrence of severe cases that are refractory to the treatment due to the low bioavailability and efficiency of some drugs in advanced lesions. Some patients of our study took more than a year to obtain a correct diagnosis and initiate adequate treatment.

In our cases, treatment dropouts was high and they were likely related to delayed clinical responses and the prolonged treatment times. Recurrence was also frequent [56] and prevailed in patients that had undergone surgery, especially in the eumycetoma group [38]. The reasons are unknown, but may be likely due to the existence of undiagnosed subclinical lesions fungal defence mechanisms against antifungal drugs or incomplete surgical procedures. It is interesting to note that in case 2 (Table 2), the patient was considered clinically cured, but presented recurrence at the eighth year of follow-up [38]. In this case, however, exogenous reinfection cannot be ruled out. We did not observe a relationship between recurrence and a specific etiologic agent.

In rarely cases, mycetoma can spread along the lymphatics to the regional lymph node [6,68]. Few blood-spread mycetoma cases [7,16,69,70,71,72] and deaths related to the infection were reported [4,9,70], but they were not observed in our study.

Although with few cases, this study, highlights the wide spectrum of clinical manifestations of mycetoma, such as localized lesions, bone disease, worsening with pregnancy, recurrence and amputation cases. We also emphasize the challenges to treat and control this neglected disease. The accurate management of each case requires multiple experts including clinicians, surgeons, microbiologists, radiologists working together to assess the best therapeutic approach, which includes a prolonged treatment followed by a long follow up after achieving clinical cure. Rehabilitation is necessary in cases of deformity and amputation, unacceptable sequelae in the 21th century.
